# Factors Affecting the Direct and Indirect Performance of Infection Control for Centrally Inserted Central Catheters Among ICU Nurses

**DOI:** 10.3390/healthcare13090988

**Published:** 2025-04-24

**Authors:** Yoonjeong Park, Seunghye Choi

**Affiliations:** 1 Intensive Care Nursing Department, Gachon University Gil Medical Center, 21, Namdong-daero, Yeonsu-gu, Incheon 21565, Republic of Korea; ua0924@gachon.ac.kr; 2 College of Nursing, Gachon University, 191 Hambakmoero, Yeonsu-gu, Incheon 21936, Republic of Korea

**Keywords:** central venous catheter, nursing professionalism, organizational culture, infection control performance, ICU nurses

## Abstract

Background/Objectives: This descriptive study investigated the influence of intensive care unit (ICU) nurses’ knowledge and perception of the importance of patient safety management, nursing professionalism, and infection control organizational culture on the direct and indirect performance of centrally inserted central catheter (CICC) infection control. Direct performance encompasses immediate infection control interventions administered to patients, whereas indirect performance constitutes physician communication and documentation protocols. Methods: A structured questionnaire was used to survey 176 ICU nurses from a tertiary hospital in Korea. Results: There were no significant differences in CICC infection control performance according to participants’ general characteristics. The direct performance of CICC infection control showed a significant positive correlation with clinical experience (*p* = 0.006), ICU work experience (*p* = 0.020), the perception of the importance of patient safety management (*p* < 0.001), nursing professionalism (*p* < 0.001), and infection control organizational culture (*p* < 0.001). The indirect performance of CICC infection control did not show any significant correlation with participants’ general characteristics; however, it showed significant positive correlations with the perception of the importance of patient safety management (*p* < 0.001), nursing professionalism (*p* < 0.001), and infection control organizational culture (*p* < 0.001). The factors affecting the direct performance of CICC infection control were the perception of the importance of patient safety management and infection control organizational culture. The perception of the importance of patient safety management affected the indirect performance of CICC infection control. Conclusions: To enhance overall infection control performance among ICU nurses, it is crucial to raise the perception of the importance of patient safety management and implement systematic strategies targeting both the direct and indirect performance of CICC infection control. Healthcare institutions should establish more detailed guidelines distinguishing between direct and indirect performance of CICC infection control and continuously educate ICU nurses on the importance of compliance with both aspects.

## 1. Introduction

According to the 2020–2021 surveillance data from the Korean National Healthcare-associated Infections Surveillance System (KONIS), the device-associated infection rate of central-line-associated bloodstream infections (CLABSI) was 2.21 per 1000 device days (DDs) [1]. A total of 4435 cases of healthcare-associated infections in intensive care units (ICUs) are reported annually in South Korea [1]. Among these, bloodstream infections are the most common, of which approximately 87.0% are associated with centrally inserted central catheters (CICCs) [1]. CICCs are medical devices frequently used in ICUs for fluid administration, medication, parenteral nutrition, transfusion, and central venous pressure monitoring [2].

CICC-associated bloodstream infections (CICC-BSIs) increase hospital stay and mortality rates [3]. To prevent CICC-BSIs, the Centers for Disease Control and Prevention (CDC) issued comprehensive prevention guidelines in the form of bundles rather than a single nursing procedure [4]. This bundle includes standardized components, such as CICC insertion site selection, infection control guidelines during insertion, types of disinfectants, and the timing of catheter removal [4]. Most CICC-BSIs can be prevented using appropriate aseptic techniques, management, and adherence to the bundle [5]. ICU nurses’ knowledge of infection control is reportedly a significant factor influencing bloodstream infection prevention [6]. However, existing studies have shown inconsistent relationships between knowledge and performance [6,7,8], highlighting the need to comprehensively identify factors beyond infection control knowledge that affect the performance of CICC infection control.

Previous studies have reported various factors influencing nurses’ performance, including individual characteristics such as the perception of the importance of patient safety management [9] and nursing professionalism [10], as well as the impact of organizational culture [11]. The perception of the importance of patient safety management is crucial for ICU nurses to consistently perform patient safety management activities and adhere to standard precaution guidelines even in urgent situations [9,12]. Nursing professionalism is a structured perspective on professional consciousness regarding nursing and nurses and is directly linked to the practice of nursing activities [13]. Meanwhile, a safe environment can enhance patient-safety-related nursing activities [12]. Specifically, organizational culture is known to modify the performance of healthcare workers and influence infection prevention and control performance [14]. Close interactions within multiple management systems influenced by organizational culture are necessary for effective infection control [11].

As the guidelines for preventing CICC-BSIs are composed of a bundle [4], the performance of CICC infection control can be categorized into direct and indirect activities (e.g., cognitive workload and reporting tasks to physicians) [15,16]. A previous study found that nurses perceived indirect nursing activities, such as reporting and decision making with physicians, as not being part of their core responsibilities and as tasks blurred with those of other professions [17]. Role confusion and task ambiguity can weaken nurses’ competencies [18]. Therefore, it is necessary to closely examine CICC infection control by dividing it into direct performance, performed directly on patients, and indirect performance, which involves reporting to physicians, in order to provide a more systematic approach and high-quality nursing care.

This study investigated the knowledge of CICC infection control, perception of the importance of patient safety management, nursing professionalism, infection control organizational culture, and the direct and indirect performance of CICC infection control. It attempted to identify the factors affecting direct and indirect performance. Specifically, we hypothesized that although knowledge of CICC infection control, perception of the importance of patient safety management, nursing professionalism, and infection control organizational culture would serve as determinants influencing nurses’ CICC infection control performance, the magnitude of their impact would vary between direct and indirect performance measures.

## 2. Materials and Methods

### 2.1. Study Design

This study is a descriptive survey using a structured questionnaire.

### 2.2. Participants

ICU nurses working at a tertiary hospital in the city were recruited through convenience sampling. The inclusion criteria were ICU nurses (internal medicine, surgery, neurosurgery, cardiovascular, trauma, and emergency) who provided direct patient care, had at least six months of work experience at the hospital, and understood the study purpose and voluntarily agreed to participate. The exclusion criteria were ICU nurses with less than six months of work experience or head nurses with limited direct patient care responsibilities.

The required sample size was calculated using the G*Power 3.1.9.7 program [19], with a medium effect size of 0.15 [12], a significance level of 0.05, a power of 0.90, and 15 independent variables, resulting in a required sample size of 171 participants. Considering a 10% dropout rate, the final sample size was 190.

### 2.3. Instruments

The structured questionnaire consisted of 97 questions on general characteristics (10), knowledge of CICC infection control (15), perception of the importance of patient safety management (21), nursing professionalism (29), infection control organization culture (10), and performance of CICC infection control (12). Participants’ general characteristics included sex, age, highest level of education, position, department, total clinical experience, ICU work experience, number of patients in charge, infection control education experience, and presence of CICC infection control guidelines.

#### 2.3.1. Knowledge of CICC Infection Control

The tool for assessing knowledge of CICC infection control was adapted and refined using prior tools [7,20], with modifications to reflect actual clinical terminology. The items were reviewed for content validity (CVI) by a nurse working in the infection control department with > 20 years of clinical experience, two advanced-practice nurses in infection control, and two nursing professors with ICU experience. The final expert CVI was 0.97. The tool consists of three response categories: “correct,” “incorrect,” and “don’t know.” Correct answers were scored 1 point, while “incorrect” and “don’t know” were scored 0 points. The reliability of the previous study was determined using the Kuder–Richardson formula, 20 = 0.50 [20], which indicates a relatively low reliability. In this study, the reliability of the tool, determined using the Kuder–Richardson formula, was 20 = 0.59, which also shows relatively low reliability.

#### 2.3.2. Perception of the Importance of Patient Safety Management

The perception of the importance of patient safety management was measured using a previously developed tool [9] that consists of 21 items divided into four subdomains: seven items on interest in patient safety management, five on confidence in patient safety management, five on willingness to engage in patient safety management, and four on awareness of patient safety management. Each item is rated on a 5-point Likert scale ranging from 1 (“Not at all”) to 5 (“Very much so”), with higher scores indicating a higher perception of the importance of patient safety management. At the time of development, the reliability of the measurement tool, measured using Cronbach’s α, was 0.86 [9]. In this study, Cronbach’s α was 0.97.

#### 2.3.3. Nursing Professionalism

Professionalism was assessed using a previously developed tool [13] that consists of 29 items divided into five subdomains: 9 items on professional self-concept, 8 on social recognition, 5 on nursing professionalism, 4 on nursing practice roles, and 3 on nursing autonomy. Each item is rated on a 5-point Likert scale ranging from 1 (“Not at all”) to 5 (“Very much so”), with higher scores indicating a well-established level of nursing professionalism. At the time of development, the reliability of the tool, measured using Cronbach’s α, was 0.92 [13]. In this study, Cronbach’s α was 0.94.

#### 2.3.4. Infection Control Organization Culture

Infection control organizational culture was measured using an existing tool, which is a modified and supplemented version by Moon and Jang [21] based on the patient safety culture measurement tool [22]. It consists of 10 items, each rated on a 7-point Likert scale ranging from 1 (“Strongly disagree”) to 7 (“Strongly agree”). In the previous study, the reliability of the tool, measured using Cronbach’s α, was 0.85 [21]. In this study, Cronbach’s α was 0.87.

#### 2.3.5. Performance of CICC Infection Control

The performance of CICC infection control was measured using self-reported survey data collected through a previous tool [20] modified from the Standard Precautions for Healthcare-associated Infections [23] and the CICC Infection Control Performance Tool [7]. Moreover, we revised the text to include easily understandable terms for study participants and terminology commonly used in actual hospital settings. CVI was evaluated by experts (a nurse working in the infection control department with > 20 years of clinical experience, two advanced-practice nurses in infection control, and two nursing professors with ICU experience). The final expert CVI was 0.97. The tool comprises 12 items, rated on a 5-point Likert scale ranging from 1 (“Never”) to 5 (“Always”). Higher scores indicate higher levels of performance.

The direct performance of CICC infection control refers to nursing activities that involve directly performing infection control measures in patients [6]; it accounted for 9 out of the 12 items (comply with aseptic technique when disinfecting and manipulating CICC; replace dressing at insertion site when it becomes wet, loose, or soiled; use sterile gauze for dressing at insertion site when patient sweats heavily or when bleeding or fluid leakage occurs; replace gauze dressing every 2 days; replace transparent dressing (Tegaderm) every 7 days; prepare for maximal sterile barrier precautions during central line insertion using cap, mask, sterile gown, sterile gloves, and full-body sterile drape; unless contraindicated, prepare skin antisepsis with >2% chlorhexidine with alcohol before catheter insertion and during dressing changes; prepare to use central venous catheter with minimum number of ports and lumens essential for patient management; and thoroughly disinfect injection ports, catheter hubs, and needleless connectors with alcohol, 2% chlorhexidine, or povidone antiseptic for 3–15 s before and after use and allow to dry completely before medication administration). Indirect performance refers to activities related to reporting to physicians, as indicated by the “report” items in the CICC infection control performance tool; it consisted of three items (in adults, inform physician to avoid femoral vein insertion if possible when attempting to insert central venous catheter into femoral vein; inform physician to remove central venous catheter promptly when no longer needed; and inform physician to wait until antiseptic solution has completely dried before inserting central venous catheter). In a previous study, Cronbach’s α was 0.79 [7]. In this study, the overall Cronbach’s α for the tool was 0.85, with a 0.90 Cronbach’s α for direct performance and 0.78 for indirect performance.

### 2.4. Ethical Consideration

This study was conducted after obtaining approval from the Institutional Review Board (IRB) of the relevant institution (IRB No. GAIRB2024-246). The participants who completed the questionnaire were provided a small token of appreciation in the form of a mobile gift voucher.

### 2.5. Data Collection

Data were collected using a mobile questionnaire following approval from the IRB and permission from the Nursing Headquarters from 27 September 2024 to 5 November 2024. The approved departments were visited to request cooperation from the department heads, and recruitment notices were posted on departmental bulletin boards. The notices included the study purpose and method as well as a QR code for the mobile questionnaire. It was stated that the study results would be used solely for research purposes. Before proceeding with the actual mobile data collection, we conducted preliminary testing to ensure there were no issues with font size and survey layout that might affect readability and response completion. On the first page of the mobile questionnaire, an information sheet about the study and a consent form were provided. The participants could proceed with the survey only after reading and agreeing to the terms. Participants self-reported and completed the mobile questionnaire directly. The questionnaire could not be submitted if any items remained unanswered. Participants were informed they could withdraw from the survey at any time without any disadvantages if they no longer wished to participate. In total, 190 responses were collected. After excluding 14 responses from individuals who did not consent to participate (*n* = 1), were not eligible as study participants (*n* = 6), or provided incomplete or insincere answers (*n* = 7), 176 responses were used for data analysis.

### 2.6. Data Analysis

The collected data were analyzed using the SPSS WIN 30.0 program (IBM Corp., Armonk, NY, USA, 2021). Participants’ general characteristics were analyzed using frequency, percentage, mean, and standard deviation. Nurses’ knowledge of CICC infection control, perception of the importance of patient safety management, nursing professionalism, infection control organizational culture, and direct and indirect performance of CICC infection control were analyzed using means and standard deviations. Differences in the direct and indirect performance of CICC infection control according to participants’ general characteristics were analyzed using the Mann–Whitney test, ANOVA, or Kruskal–Wallis test, depending on whether normality was satisfied after the Kolmogorov–Smirnov test. Post hoc analysis was conducted using Scheffe’s test. To examine multicollinearity between independent variables and determine variable selection for the multiple regression model, correlation coefficients were computed before the analysis. The correlation between knowledge of CICC infection control, perception of the importance of patient safety management, nursing professionalism, infection control organization culture, and direct and indirect performance of CICC infection control were analyzed using Pearson’s correlation coefficients. Prior to conducting multiple regression analysis, multicollinearity testing was performed. Multicollinearity diagnostics revealed no concerning relationships among the independent variables, with all tolerance values exceeding 0.1 and variance inflation factors (VIFs) remaining below 10. Additionally, the Durbin–Watson statistic approximated 2, indicating no autocorrelation in the residuals. Multiple regression analysis was used to identify factors influencing the direct and indirect performance of CICC infection control.

## 3. Results

### 3.1. Participants’ General Characteristics

Of the study participants, 140 (79.5%) were women. Their ages ranged from 22 to 43 years, with an average age of 28.04 ± 4.36 years. The majority were general nurses (*n* = 162, 92%). Among these departments, 57 (32.4%) worked in the medical ICU, 35 (19.9%) in the emergency ICU, 34 (19.3%) in the surgical ICU, 30 (17%) in the trauma ICU, and 20 (11.4%) in the cardiovascular ICU. The number of patients assigned per nurse ranged from 1 to 3, with an average of 2.40 ± 0.52 patients. A total of 140 nurses (79.5%) reported having received infection control education, and 135 (76.7%) responded that their department had infection control guidelines (Table 1).

### 3.2. Level of Knowledge of CICC Infection Control, Perception of the Importance of Patient Safety Management, Nursing Professionalism, Infection Control Organization Culture, and Direct and Indirect Performance of CICC Infection Control

Knowledge of CICC infection management among ICU nurses showed an average correct response rate of 78.8% (Table 2). Among the subdomains of perception of the importance of patient safety management, awareness of patient safety management showed the highest score at 4.57 ± 0.55 points, followed by willingness for patient safety management (4.32 ± 0.60) and interest in patient safety management (4.31 ± 0.61). Confidence in patient safety management had the lowest subdomain score at 4.24 ± 0.64 points (Table 2).

The analysis of nursing professionalism subdomains revealed that nursing practice role demonstrated the highest score at 4.04 ± 0.61 points, and autonomy of nursing had the lowest subdomain score at 2.92 ± 0.90 points (Table 2).

The performance of CICC infection control was divided into direct and indirect tasks, with direct performance averaging 4.71 ± 0.41 points and indirect performance averaging 3.96 ± 0.90 points (Table 2).

### 3.3. Differences in Direct and Indirect Performance of CICC Infection Control According to Participants’ General Characteristics

No variables showed significant differences in participants’ general characteristics nor the direct or indirect performance of CICC infection control (Table 3).

### 3.4. Correlation Between Knowledge of CICC Infection Control, Perception of the Importance of Patient Safety Management, Nursing Professionalism, Infection Control Organization Culture, and Direct and Indirect Performance of CICC Infection Control

The direct performance of CICC infection control was significantly positively correlated with the perception of the importance of patient safety management (*r* = 0.59, *p* < 0.001), nursing professionalism (*r* = 0.33, *p* < 0.001), and infection control organizational culture (*r* = 0.51, *p* < 0.001). Indirect performance showed a statistically significant positive correlation with the perception of the importance of patient safety management (*r* = 0.41, *p* < 0.001), nursing professionalism (*r* = 0.33, *p* < 0.001), and infection control organizational culture (*r* = 0.33, *p* < 0.001) (Table 4).

There was a significant positive correlation between the perception of the importance of patient safety management and nursing professionalism (*r* = 0.57, *p* < 0.001) as well as between the perception of the importance of patient safety management and infection control organizational culture (*r* = 0.71, *p* < 0.001). Nursing professionalism and infection control organizational culture (*r* = 0.63, *p* < 0.001) also showed a significant positive correlation (Table 4).

### 3.5. Factors Affecting the Performance of CICC Infection Control

#### 3.5.1. Direct Performance

A multiple regression analysis was conducted to analyze the factors influencing participants’ direct performance of CICC infection control. Work experience, perception of the importance of patient safety management, nursing professionalism, and infection control organizational culture showed significant correlations with direct performance and demonstrated significant relationships in the univariate analysis. These were included as independent variables and analyzed using the Enter method. Although clinical experience was also significant in the correlation analysis, it exhibited multicollinearity with ICU work experience, which was used as an independent variable.

The perception of the importance of patient safety management and the organizational culture of infection control significantly affect direct performance. Direct performance was higher when the perception of the importance of patient safety management was greater (β = 0.44, *p* < 0.001) and when the organizational culture of infection control was more positive (β = 0.22, *p* < 0.020). Among the influencing factors, the perception of the importance of patient safety management had the greatest impact; the explanatory power of these influencing factors was 35.5% (*F* = 25.12, *p* < 0.001) (Table 5).

#### 3.5.2. Indirect Performance

Perceptions of the importance of patient safety management, nursing professionalism, and the organizational culture of infection control showed significant correlations with indirect performance and significant relationships in the univariate analysis. These were included as independent variables and analyzed using the Enter method.

The perception of the importance of patient safety management most significantly influenced indirect performance. The higher the perception of the importance of patient safety management (β = 0.33, *p* = 0.002), the higher the indirect performance. The explanatory power of this variable for indirect performance was 16.9% (*F* = 12.88, *p* < 0.001) (Table 6).

## 4. Discussion

This study analyzed ICU nurses’ levels of knowledge about CICC infection control, perception of the importance of patient safety management, nursing professionalism, infection control organizational culture, and performance of CICC infection control. It also identified the factors influencing the direct and indirect performance of CICC infection control.

The overall score for the performance of CICC infection control in this study is similar to that in previous studies using the same tool [20]. Among the direct performance items, “Gauze dressings are replaced every two days” and “Dressings using Tegaderm are replaced every seven days” scored the highest. Previous studies have shown that dressings exhibit the highest performance [6]. In this study, the highest-scoring item in the knowledge of CICC infection control category was also related to dressings. A previous study also reported a high score for the item “Transparent dressings for CICCs are replaced within 5–7 days” [6]. The item “Prepare CICCs with the minimum number of ports and lumens necessary for patient treatment” had the lowest performance score, which is consistent with the results of previous studies [6,20]. Although having minimum ports is advantageous from an infection control perspective, the number of ports available for drug administration is more important in real-world ICU settings where critical medications are continuously infused into critically ill patients; therefore, this activity may have been given a lower priority in the performance of CICC infection control. In this study, there were no statistically significant differences in CICC infection control performance according to participants’ general characteristics (sex and education level). This finding showed some discrepancies with previous research. According to the previous study [7], while there were no sex differences in CICC infection control performance, significant differences were reported based on educational levels. The reason for the different results from previous studies may be that in this study, participants with a bachelor’s degree accounted for the majority (87.5%) of the sample, which may have resulted in no statistical differences. Therefore, further research with a larger sample size is needed to validate these findings.

Among the indirect performance items, “Notify the physician immediately to remove the CICC if it is no longer needed” scored the highest, whereas “In cases of adult patients, notify the physician to avoid inserting the CICC into the femoral vein whenever possible” scored the lowest. This result is similar to that of previous studies [20]. Items related to CICC insertion might have been perceived as being under the physician’s judgment, leading to lower performance scores. In this study, direct performance was higher than indirect performance. This is consistent with previous studies showing that nurses tend not to perceive indirect performance as their unique nursing duty [17], and that role confusion weakens nursing competency [18]. The longer the CICC remains inserted, the higher the risk of catheter-related bloodstream infections [24]; the insertion site is also known to influence the risk of bloodstream infections [25]. Therefore, actively notifying physicians is an essential element of infection control practice among nurses. By reducing work-related confusion among nurses, this will enhance communication with medical staff and improve the overall performance of infection control competency.

When analyzing the correlations of knowledge of CICC infection control, perception of the importance of patient safety management, nursing professionalism, and infection control organizational culture with the direct and indirect performance of infection control, knowledge of CICC infection control alone did not show a significant correlation. Previous studies have reported inconsistent results. Some studies have reported a positive correlation between knowledge of CICC infection control and performance [6]. However, according to a previous study, only 5% of ICU nurses reported complete adherence to evidence-based practices for preventing central-line-associated bloodstream infections [26]. Additionally, some studies have reported a gap between the high average knowledge levels of ICU nurses and their actual performance [8]. Therefore, various strategies that go beyond simply providing knowledge must be established to improve the actual performance of CICC infection control among ICU nurses. Furthermore, since the reliability of the knowledge measurement tool in this study was relatively low, future studies should consider improving the tool by increasing the number of items or revising the content to better assess CICC infection control knowledge among ICU nurses.

The perception of the importance of patient safety management affects both direct and indirect performance, making it the most critical factor influencing CICC infection control performance. This finding aligns with that of previous studies indicating that it affects nurses’ adherence to standard precautions [27,28]. The simulation-based training about patient safety management significantly improved patient safety competency among ICU nurses [29]. Therefore, strategies are needed to establish ICU nurses’ awareness regarding the importance of patient safety management and to enable them to perform safety and infection control tasks.

Infection control organizational culture affects direct performance but not indirect performance. According to a previous study, indirect nursing activities include documentation, input, and reporting [30]. In this study, all items related to indirect performance fell under the reporting category. The organizational culture did not affect indirect performance, which includes communication with other healthcare professionals, because the organizational culture instrument was limited to nursing departments rather than the entire hospital organization, and indirect performance activities were relatively less monitored, potentially weakening the influence of organizational culture. Moreover, since the explanatory power for indirect performance was low, further research is needed to explore additional variables that influence indirect performance. Previous studies have shown that leaders’ roles in healthcare-associated infection prevention activities include communicating with staff, overcoming barriers, and inspiring their staff [31]. Therefore, managers and supervisors within organizations need to provide positive feedback to staff to raise awareness of the importance of infection control. Additionally, fostering a culture that accepts and implements improvement suggestions related to infection control from less-experienced nurses can help establish a desirable organizational culture for infection control.

Nursing professionalism did not affect the direct or indirect performance of CICC infection control. This is inconsistent with previous studies reporting that professionalism directly affects patient safety competency [32]. This may reflect that infection control in clinical settings is guided more by standardized protocols rather than nurses’ professional judgment, or it could be attributed to the fact that the study participants had an average clinical experience of less than 5 years, suggesting their nursing professionalism may not yet have been fully developed. Further multicenter studies are required to confirm these findings.

Considering the association between CICC infection control performance and organizational culture found in this study, not only establishing individual nurses’ awareness of the importance of patient safety management but also fostering an infection control organizational culture could be important elements in improving nurses’ CICC infection control performance. It is necessary to educate nurses by dividing CICC infection control performance into direct and indirect nursing activities and clearly define the scope of indirect nursing activities at the hospital level. Organizational efforts are needed to develop tailored educational programs that enable ICU nurses to demonstrate their infection control competencies.

The limitations of this study were as follows. First, this study was conducted with a small sample of ICU nurses working at a single tertiary hospital in a specific region and used a convenience sampling method; therefore, the findings cannot be generalized to all ICU nurses. Second, the average years of experience of the participants was less than five years, which requires caution when interpreting the results. Third, this study was conducted at a specific point in time, and changes in infection control performance over time were not considered. Fourth, this study investigated CICC infection control performance through self-reporting methods, which may differ from actual performance. Finally, CICC-BSIs can be strictly divided into catheter-related bloodstream infections (CRBSI) and central-line-associated bloodstream infections (CLABSI). CLABSI is more commonly used for hospital infection control; therefore, this study focused on investigating CLABSI. However, future studies need to carefully examine and distinguish between CRBSI and CLABSI [33].

## Figures and Tables

**Table 1:** Participants’ general characteristics (*N* = 176).

Variable	Category	*N* (%)	M ± SD (Min, Max)
Sex	Female	140 (79.5)	
	Male	36 (20.5)	
Age (year)			28.04 ± 4.36 (22, 43)
Highest level of education (degree)	Associate	15 (8.5)	
	Bachelor	154 (87.5)	
	Master	7 (4)	
Position	General nurse	162 (92)	
	Charge nurse	14 (8)	
Working department	Medical	57 (32.4)	
	Surgical	34 (19.3)	
	Cardiovascular	20 (11.4)	
	Emergency	35 (19.9)	
	Trauma	30 (17)	
Clinical experience (year)			4.62 ± 4.27 (0.5, 23.33)
ICU work experience (year)			4.11 ± 3.84 (0.25, 23.33)
Number of patients in charge of			2.40 ± 0.52 (1, 3)
Infection control education experience	Yes	140 (79.5)	
	No	36 (20.5)	
Presence of CICC infection control guidelines	Yes	135 (76.7)	
	No	41 (23.3)	

M = mean; SD = standard deviation; ICU = intensive care unit; CICC = centrally inserted central catheter.

**Table 2:** Participants’ level of knowledge of CICC infection control, perception of importance on patient safety management, nursing professionalism, infection control organization culture, and direct and indirect performance of CICC infection control (*N* = 176).

Variable (No. of Item)	M ± SD	Min	Max	Item Mean
**Knowledge of CICC infection control (15)**	78.80 ± 11.80	27.00	100	
**Perception of importance on patient safety management (21)**	91.22 ± 11.54	63.00	105.00	4.36 ± 0.54
Interest (7)	30.14 ± 4.24	20.00	35.00	4.31 ± 0.61
Confidence (5)	21.22 ± 3.19	14.00	25.00	4.24 ± 0.64
Willingness (5)	21.59 ± 3.00	15.00	25.00	4.32 ± 0.60
Awareness (4)	18.28 ± 2.20	11.00	20.00	4.57 ± 0.55
**Nursing professionalism** (29)	108.70 ± 16.21	61.00	144.00	3.69 ± 0.49
Professional self-concept (9)	35.66 ± 5.82	19.00	45.00	3.96 ± 0.65
Social recognition (8)	28.23 ± 6.63	9.00	40.00	3.53 ± 0.83
Nursing professionalism (5)	19.91 ± 3.00	9.00	25.00	3.98 ± 0.60
Nursing practice role (4)	16.15 ± 2.43	10.00	20.00	4.04 ± 0.61
Autonomy of nursing (3)	8.75 ± 2.69	3.00	15.00	2.92 ± 0.90
**Infection control organization culture** (10)	56.33 ± 8.42	39.00	70.00	5.63 ± 0.84
**Performance of CICC infection control** (12)	54.31 ± 5.29	36.00	60.00	4.53 ± 0.44
Direct performance (9)	42.43 ± 3.72	27.00	45.00	4.71 ± 0.41
Indirect performance (3)	11.88 ± 2.69	3.00	15.00	3.96 ± 0.90

M = mean; SD = standard deviation; CICC = centrally inserted central catheter.

**Table 3:** Differences in direct and indirect performance of CICC infection control according to participants’ general characteristics (*N* = 176).

Variable	Category	Performance of CICC Infection Control			
		Direct Performance		Indirect Performance	
		M ± SD	*F*/*Z* (*p*)	M ± SD	*F*/*Z* (*p*)
Sex	Female	42.39 ± 3.85	−0.32 * (0.747)	11.94 ± 2.71	−0.75 * (0.454)
	Male	42.61 ± 3.17		11.64 ± 2.61	
Highest level of education (degree)	Associate	42.33 ± 3.60	1.67 ^†^ (0.435)	12.73 ± 1.94	1.61 ^†^ (0.447)
	Bachelor	42.36 ± 3.80		11.78 ± 2.73	
	Master	44.29 ± 0.95		12.29 ± 3.15	
Position	General nurse	42.27 ± 3.82	−1.79 * (0.073)	11.87 ± 2.70	−0.16 * (0.875)
	Charge nurse	44.36 ± 0.74		12.00 ± 2.69	
Department	Internal	42.40 ± 4.03	0.09 (0.911)	11.87 ± 2.92	0.01 (0.989)
	Surgical	42.24 ± 3.69		11.94 ± 0.98	
	Emergency or trauma	42.57 ± 3.38		11.86 ± 2.76	
Infection control education experience	Yes	42.64 ± 3.61	−1.57 * (0.116)	11.96 ± 2.70	−0.96 * (0.338)
	No	41.64 ± 4.06		11.56 ± 2.65	
Presence of CICC infection control guidelines	Yes	42.60 ± 3.19	−0.09 * (0.924)	12.07 ± 2.56	−1.44 * (0.150)
	No	41.88 ± 5.09		11.27 ± 3.03	

* = Mann–Whitney; ^†^ = Kruskal–Wallis; M = mean; SD = standard deviation; *Z* = standardized statistic in Mann–Whitney or Kruskal–Wallis testing; *F* = test statistic for ANOVA; CICC = centrally inserted central catheter.

**Table 4:** Correlation between knowledge of CICC infection control, perception of importance on patient safety management, nursing professionalism, infection control organization culture, and direct and indirect performance of CICC infection control (*N* = 176).

	(1)	(2)	(3)	(4)	(5)	(6)
	*r*(*p*)	*r*(*p*)	*r*(*p*)	*r*(*p*)	*r*(*p*)	*r*(*p*)
Knowledge (1)	1					
Perception (2)	−0.020 (0.791)	1				
Nursing professionalism (3)	−0.174 (0.021)	0.569 (<0.001)	1			
Infection control organization culture (4)	−0.073 (0.338)	0.714 (<0.001)	0.632 (<0.001)	1		
Direct performance (5)	0.104 (0.169)	0.585 (<0.001)	0.332 (<0.001)	0.508 (<0.001)	1	
Indirect performance (6)	−0.018 (0.815)	0.412 (<0.001)	0.331 (<0.001)	0.331 (<0.001)	0.350 (<0.001)	1

*r* =correlation coefficient; CICC = centrally inserted central catheter.

**Table 5:** Factors affecting the direct performance of CICC infection control (*N* = 176).

	B	SE	β	*t*	*p*
ICU work experience	0.089	0.06	0.09	1.47	0.145
Perception of importance on patient safety management	0.143	0.03	0.44	4.87	<0.001
Nursing professionalism	−0.013	0.02	−0.06	−0.69	0.491
Infection control organization culture	0.098	0.04	0.22	2.36	0.020
*R*^2^ = 0.370, Adj *R*^2^ = 0.355, *F* = 25.12, *f*^2^ = 0.55, *p* < 0.001					

SE = standard error; *f*^2^ = effect size.

**Table 6:** Factors affecting the indirect performance of CICC infection control (*N* = 176).

	B	SE	β	*t*	*p*
Perception of importance on patient safety management	0.076	0.02	0.33	3.22	0.002
Nursing professionalism	0.023	0.02	0.14	1.52	0.131
Infection control organization culture	0.004	0.03	0.01	0.11	0.911
*R*^2^ = 0.183, Adj *R*^2^ = 0.169, *F* = 12.88, *f*^2^ = 0.23, *p* < 0.001					

SE = standard error; *f*^2^ = effect size.

## Data Availability

The datasets generated and/or analyzed during the current study are not publicly available in order to protect the participants but are available from the corresponding author upon reasonable request.

## Abbreviations

The following abbreviations are used in this manuscript:

KONIS	Korean National Healthcare-associated Infections Surveillance System
DD	Device days
ICU	Intensive care unit
CICC	Centrally inserted central catheter
CICC-BSI	CICC-associated bloodstream infections
CDC	Centers for Disease Control and Prevention
CVI	Content validity index
IRB	Institutional Review Board
ANOVA	Analysis of variance
M	Mean
SD	Standard deviation
SE	Standard error
VIF	Variance inflation factor
